# A New Insight Into Pollen Release and Presentation in Actinidiaceae Plants: The Case Study of Dioecious *Actinidia arguta*

**DOI:** 10.1002/ece3.73247

**Published:** 2026-04-03

**Authors:** Kexin Sun, Xueying Yang, Shanlin Yang, Rongrong Cui, Yan Ji, Yuting Hu, Renjie Li, Dingxu Jin

**Affiliations:** ^1^ College of Landscape Architecture Changchun University Changchun China

**Keywords:** dioecy, pollination mechanism, reproductive strategy, sequential pollen presentation, sexual dimorphism

## Abstract

Sequential pollen presentation is a reproductive strategy adopted by plants to enhance the efficiency and accuracy of pollen export and receipt, as well as to promote outcrossing. *Actinidia arguta* exhibits a typical characteristic of sequential pollen presentation. In this study, indoor and field observation methods were employed to conduct a tracking investigation on the anther dehiscence mode, pollen presentation strategy of male, and the floral characteristics and pollination traits of both female and male. The results showed that the dark‐colored structures surrounding the ovary in the female flower are sterile staminodes. The female flowers possess multi‐lobed stigmas arranged radially. The anthers of male are black, and the petals of female are significantly larger than male. The anther dehiscence mode of male *A. arguta* is “unzipping” longitudinal dehiscence for pollen release, and its pollen presentation strategy is sequential presentation. The peak period of pollen release effectively overlaps with the periods of pollen viability, stigma receptivity, and nectar secretion at different flowering stages. This reduces pollen waste and promotes outcrossing pollination. Male *A. arguta* produces a large amount of pollen and secretes more nectar than female. This attracts more pollinating insects, indicating that *A. arguta* is a typical insect‐pollinated plant. *Apis cerana* and *Bombus* are the main pollinators, and pollination is accomplished by relying on these pollinating insects to transfer pollen from male to the stigmas of female. Exploring the pollination mechanism and characteristics of *A. arguta* pollen deepens the scientific understanding of its pollination process.

## Graphical abstract

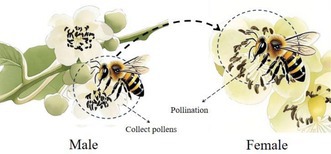

## 1 Introduction

The adaptive evolution of angiosperms to different pollinators (insects, birds, etc.) and different species within the same pollinator group has led to the development of a wide variety of floral characteristics. The evolution of floral characteristics is the result of adaptation to pollinator insects, and adaptation to pollination is the fundamental driving force behind the evolution of floral characteristics (Meng et al. 2024). Differences in floral structure, corolla size, nectar secretion, and delicate petal characteristics can attract more pollinating insects, influencing the species, quantity, and pollination behavior of pollinating insects (Ji et al. 2023). After long‐term coevolution between plants and pollinator insects, pollination behavior is closely related to the comprehensive floral characteristics of plants, which to a certain extent determines pollination efficiency and affects the reproductive fitness of plants (Meng et al. 2024; Wang and Tan 2011).

Plants control the rate of pollen release and the mode of pollen presentation through pollen presentation strategies (Song 2018; Castellanos et al. 2006). Plants attract more pollinating insects by gradually releasing pollen, reducing the pollen intake during a single visit, avoiding interference between anthers, and enhancing the reproductive success rate and adaptability of males (Li 2013; Halibunuer et al. 2022). Therefore, the gradual release strategy of pollen facilitates the process of cross‐pollination, reduces the ineffective consumption of pollen resources, and significantly enhances the pollination efficiency of plants.

Dioecy refers to where female and male flowers grow on separate plants (Broussard et al. 2021). During the long‐term evolutionary process, differences have emerged in traits such as floral phenology, floral characteristics, flower quantity, flower size, and floral rewards between male and female individuals, advertising or morphological adaptation that influence pollen transfer and receipt (Moquet et al. 2020; Ushimaru et al. 2023). Male flowers may attract more pollinators by increasing nectar secretion, while female flowers devote more resources to fruit and seed production (Sinclair et al. 2012). Male reproduction is limited to producing pollen (Zeng 2022). Understanding reproductive investment differences between male and female plants is key to understanding pollinator behavior during pollination.

*Actinidia arguta* is a dioecious perennial deciduous liana in the family Actinidiaceae. *A. arguta* is abundant in the northeastern region of China and is a wild fruit tree with high economic value and broad development prospects. Currently, domestic and international scholars' research on *A. arguta* mainly focuses on cultivation techniques and breeding. This study aims to explore the following questions: (1) The pollen presentation mechanism of *A. arguta* ; (2) Mutual adaptation between the nectar secretion strategies and pollinating insects of female and male *A. arguta* ; (3) Sexual dimorphism and reproductive cost differences between female and male *A. arguta* . This study helps to analyze the interaction between the degree of flowering of *A. arguta* , pollen and nectar, the pollinating insect's visitation behavior, providing a solid scientific basis and clear guidance for the impact of its growth and development on production practices.

## 2 Materials and Methods

### 2.1 Study Site

**FIGURE 1:**
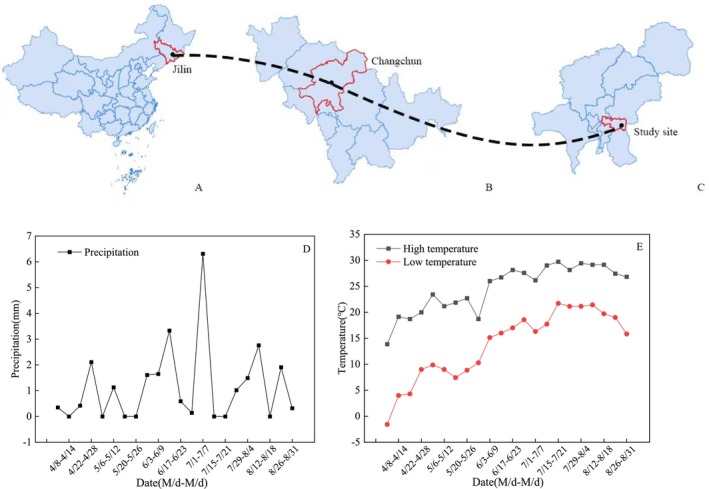
Geographical location of the study area (A–C), rainfall during the flowering period of *Actinidia arguta* (D), daily high and low temperatures (E).

This study area is located in Lianhuashan, Changchun City, Jilin Province, China (125°36′ E, 43°52′ N) (Figure 1A–C). The overall terrain in the study area is flat, with a plant population area of approximately 1500 m^2^, and it belongs to a northern temperate continental climate with large temperature differences between day and night. The average rainfall during the flowering period is 1.15 mm (Figure 1D). During the flowering period, the average daily high temperature was 24.7°C, and the average daily low temperature was 11.8°C (Figure 1E). The experimental material selected for this study was 5‐year‐old *A. arguta* .

### 2.2 Research Methods

#### 2.2.1 Flowering Phenology

On May 30, 2024, flowering phenology observations were conducted at the *A. arguta* base at four levels: population, individual, inflorescence, and single flower of both female and male plants, 60 plants with basically consistent growth were selected, from which one inflorescence was randomly chosen from each plant, resulting in 60 inflorescences. One flower bud was randomly selected from each inflorescence, totaling 60 flower buds. The sample plants, inflorescences, and flower buds were tracked and observed. Population level: The start of flowering is when 25% of the plants in the population have bloomed, the peak of flowering is when 50% or more of the plants in the population have bloomed, and the end of flowering is when 95% of the plants in the population have lost their corollas. Inflorescence and individual level: The opening of the first flower marks the start of flowering, 50% or more of the flowers in bloom marks the peak flowering period, and the complete fall of the flowers marks the end of flowering. Single flower level: regularly observe and record the flowering time and flowering period of single flowers in real time, with an observation interval of 1 day. Based on the above data, calculate the individual flowering amplitude and flowering synchrony index.

$$\text{Flowering synchronization}:S_{i}=\frac{1}{n-1}\left(\frac{1}{f_{i}}\right)\sum_{j=i}^{n}e_{j\neq i}$$

In the formula: *e*_*j*_: overlapping flowering period between individual *i* and individual *j* (dats), *f*_*j*_: duration of flowering for individual *i* (days), *n*: total number of individuals, 0 ≤ *S*_*i*_ ≤ 1 (0: no overlap in flowering period, 1: complete overlap in flowering period).

#### 2.2.2 Floral Characteristics and Flowering Process

Within the *A. arguta* population, randomly selected 10 female and 10 male plants with similar growth characteristics (height and shape) and chose one single flower from each upper, middle, and lower part of each plant. A total of 30 female flowers and 30 male flowers are labeled, and the flowering dynamics were tracked and recorded. The corolla diameter, filament and anther lengths of a single flower at different flowering stages were measured, the morphology and color of anthers were recorded, and the stigma length and color of female flowers were also recorded. Meanwhile, the flowering process of single flowers of female and male plants was recorded respectively.

**FIGURE 2:**
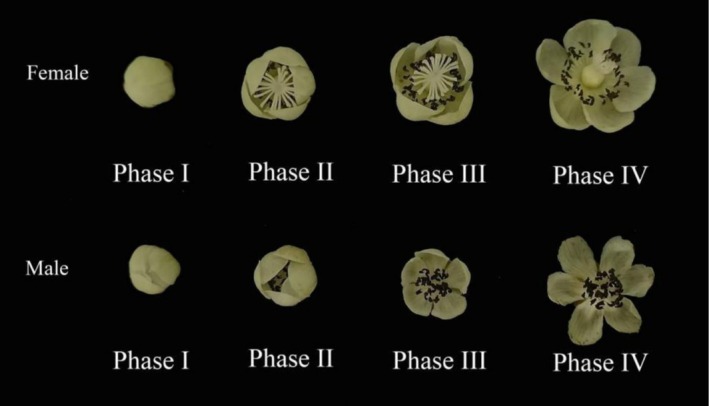
Flowering progression of female and male in *Actinidia arguta*. Phase I, initial flowering stage; Phase II, early flowering stage; Phase III, peak flowering stage; Phase IV, late flowering stage.

Based on the corolla diameter of the *A. arguta* , the flowering process of female and male is divided into four phases. Females are classified as follows: Phase I‐corolla mouth not open; Phase II‐corolla mouth opened 1–6 mm; Phase III‐corolla mouth opened 7–12 mm; Phase IV‐corolla fully opened 13–18 mm. Male are classified as follows: Phase I‐corolla mouth not open; Phase II‐corolla mouth opened 1–4 mm; Phase III‐corolla mouth opened 5–10 mm; Phase IV‐corolla fully opened 11–15 mm (Figure 2).

#### 2.2.3 Pollen Viability Testing

Within the *A. arguta* population, 10 female and 10 male flowers were randomly collected from each of the four flowering periods (divided into four flowering periods based on the Figure 2, Phase I–Phase IV). After anther detachment, a brush was used to transfer a small amount of pollen onto a microscope slide, then 1–2 drops of 0.5% TTC solution was added. The slides were put in an incubator at 20°C–25°C for 20 min and then observed under a light microscope (BM2000). Pollen viable was red, the pollen with weak viable was light red, and the flowers nonviable pollen was colorless. Five fields of view were counted for each sample, and experimental data were expressed as mean ± standard error (Pan et al. 2012).

$$\text{Pollen viability}\;\left(\%\right)=\left(\text{Stained pollen}/\text{Total pollen}\right)\times 100\%$$

#### 2.2.4 Stigma Receptivity Testing

Within the *A. arguta* population, 10 female flowers were randomly collected from each of the four flowering stages. The stigmas were removed, placed on concave slides, and dropped into the reaction solution “benzidine‐hydrogen” (1% benzidine:3% hydrogen peroxide:water = 4:11:22). A highly receptive stigma is blue with a large number of bubbles around it; a weak receptive stigma is light blue with a small number of bubbles around it; there is no color change of the stigma and no bubbles around it, which is not receptivity (Wang et al. 2024).

#### 2.2.5 Nectar Secretion Measurement

Within the *A. arguta* population, 10 inflorescences were randomly selected from each of female and male plants. The flower buds on the inflorescences (with petals starting to expand but not yet open, so that insects cannot access them) were bagged as the standard. A microcapillary tube with a diameter of 0.1 mm was used to determine the nectar secretion amount of a single flower at each of the four flowering stages. Twenty flowers were measured at each stage, and the height (*h*) of nectar in the microcapillary tube at different time points was recorded. Nectar volume (μL) per flower during different flowering stages was calculated with the following formula.

$$\text{Volume}:V={\mathrm{\pi r}}^{2}h$$

#### 2.2.6 Morphological Statistics and Observations of Anthers, Pollen Grains and Stigmas

Within the *A. arguta* population, 10 male flowers with basically consistent development at each of the four stages were randomly selected and bagged. After the single flower opened, the mature anthers that had not yet released pollen were removed, placed in a glass bottle (5 mL), stained with 1–2 drops of safranin solution, and made up to 5 mL. The glass bottle was shaken to disperse the pollen evenly, and 10 μL of the pollen solution was placed on a glass slide. The number of pollen grains on the glass slide was counted under an optical microscope to calculate the number of pollen grains per single flower, and the count was repeated six times.

Within the *A. arguta* population, 10 female flowers were randomly collected from each of the four flowering stages. The complete stigmas were taken out, fixed with 50% FAA fixative, dehydrated with gradient concentrations of ethanol (65%–75%–85%–90%–100%–100%–100%) for 15 min each time. All stigmas were dried at the critical point for 1 h and then sputtered with gold for later use. In addition, 10 male flower buds were collected at each of the four flowering stages of a single flower, and the anthers were picked to collect pollen grains, which were air‐dried and then sputtered with gold for 2.5 min. All stigmas and pollen grains were examined using a JSM‐6510 (Japan) scanning electron microscope (Changchun University). Observations included anther dehiscence mode, anther pollen release mechanisms, stigma morphology, and pollen grain morphology. The directly measured indicator is the size of the anther opening. Refer to the methods of Kang et al. and Makowski et al., the pollen presentation/exposure level of *A. arguta* was determined (Kang et al. 1993; Makowski et al. 2024). Measurements were conducted within 2 h after full bloom, with corolla diameter and anther count serving as control variables to minimize morphological interference.

(1) Operational definition: The effective extent to which pollen grains are exposed to the floral space and can be contacted and carried by pollinators after anther dehiscence. (2) Measurement criteria: A composite quantitative assessment using a 5‐grade anther dehiscence grading scale (Kang et al. 1993) and pollen exposure effectiveness ratio. The former is rated 1–5 levels based on anther dehiscence and pollen exposure degree (independent scoring by two observers, with Kappa ≥ 0.85 and mean value), while the latter is the percentage of pollen exposed per flower relative to total anther pollen (Makowski et al. 2024). (3) Control Settings: Within the same sex, the difference in corolla diameter was ≤ 0.2 cm and the number of anthers was ≤ 2.

#### 2.2.7 Pollination Characteristics

##### 2.2.7.1 Insect Visit Behavior and Identification of Pollinating Insects

From June 1 to June 6, 2024, during six sunny days in the full flowering period of *A. arguta* , five female and five male flowers were randomly selected each day, and continuous observations were conducted for 7:00–19:00 every day. The flower‐visiting behavior of flower‐visiting insects on the plants, the number of flowers visited, and the residence time on inflorescences and single flowers were carefully recorded, and the insect flower‐visiting frequency (times·flower^−1^·h^−1^) was calculated. To avoid the observer's interference with the visiting insects, all observations were conducted at a distance of more than 1 m from the insects. The visiting insects were captured using the net trapping method (Jia 2024), during the active insect period of 9:00–16:00 on sunny and windless days during the flowering season. Insects that visited and stayed on the inflorescences for more than 0.5 min were captured. The capture criteria were: the insects were in the state of visiting flowers and had contact with anthers or stigmas; only valid visiting individuals were recorded, excluding those that stayed accidentally, flew by, or did not visit flowers. The capture range covered the visiting insects within the reach of a 5‐m net pole. Insects on female and male were captured alternately every 30 min, and the number of insects appearing on female and male every 30 min was counted. After an insect completed an effective visit, it was captured with an insect net and placed in a poison bottle made of ethyl acetate to kill it. A proportion of all the visiting insects was made into dry specimens for species identification. Teacher Chen Liusheng from the Guangdong Academy of Forestry Sciences identified the insect specimens. The other proportion was observed under a Nikon SMZ‐1000 digital stereoscopic dissecting microscope to check the pollen‐carrying parts. The parts were rinsed several times with 75% alcohol, and the rinse solution was smeared on a slide and observed under an Olympus BH‐2 optical microscope to determine whether such insects carried *A. arguta* pollen, so as to judge whether they were pollinator insects.

##### 2.2.7.2 Pollination Efficiency

Within the *A. arguta* population, 10 unopened female flowers with basically consistent development were randomly selected and bagged. After the single flower opened, immediately after an insect visit, the stigma was removed and crushed on a glass slide. Using the same method as for counting pollen grains in a single flower, calculate the number of pollen grains on the stigma. This number, representing the pollen grains deposited on the stigma after a single insect visit, is recorded as A. Additionally, randomly select 10 male flowers that are at a similar stage of development and have not opened, and bagged. After a single flower has opened, stain the anthers. After a single insect visit, electric shocks were administered to visiting flowers using a portable adjustable piezoelectric shock collector (5–8 V, ≤ 0.1 mA). Each shock lasted 1–2 s, and the paralyzed insects were transferred to pre‐cooled sterile collection tubes for marking. Insects requiring observation were left undisturbed for 5–10 min prior to release (Roussel et al. 2009). Insects recovered within 5–10 min, and no behavioral abnormalities were observed post‐capture, with no cases of mortality or persistent injuries. Gently brush the pollen from the pollinator's body, ensuring no residual pollen remains on the brush. Count the number of stained pollen grains, recorded as *B*, representing the quantity of pollen grains carried away by an insect after a single visit. The calculation repeats 10 times.

$$\text{Pollination efficiency}\;\left(g\right)=A/B$$

#### 2.2.8 Artificial Pollination Experiments

Within the *A. arguta* population, flower buds are bagged before the opening of female flowers to exclude interference from pollinator insects, 50 females were selected, and 180 buds on the verge of opening were randomly marked for the following treatments: (1) Natural pollination (*n* = 60); (2) Artificial pollination (*n* = 60); (3) Bagging (*n* = 60). The artificial pollinated flowers were bagged after the pollen was manually added. When the fruits were fully mature, the fruit set rate under each treatment was counted.

### 2.3 Data Analysis

Statistical analysis of single flower corolla, filament, anther and stigma length, pollen viability, nectar secretion, visiting frequency, and pollination experiment data was conducted using SPSS 19.0 statistical software. One‐way ANOVA and independent samples *t*‐tests were used to compare differences (*p* < 0.05) in corolla, filament, anther and stigma lengths of single flowers, as well as pollen viability, nectar secretion and artificial pollination experiments across four flowering stages of female and male flowers. Independent samples *t*‐tests were conducted to compare differences in the visiting frequency of major pollinating insects (*p* < 0.05). Prior to conducting the between‐group analysis of variance (ANOVA) on the nectar secretion and the pollen viability, post hoc Tukey HSD tests were used to control for multiple comparisons. A mixed model (LMM) was employed to analyze nectar secretion and pollen viability, with plant as a random effect and phase/sex as fixed effects. Pearson correlation analysis and linear regression analysis of anther diameter and number of pollen grains single flower. Experimental data are presented as mean ± standard error. All figures were drawn using Origin 2021 software.

## 3 Results

### 3.1 Phenological Characteristics

#### 3.1.1 Flowering Phenology

**FIGURE 3:**
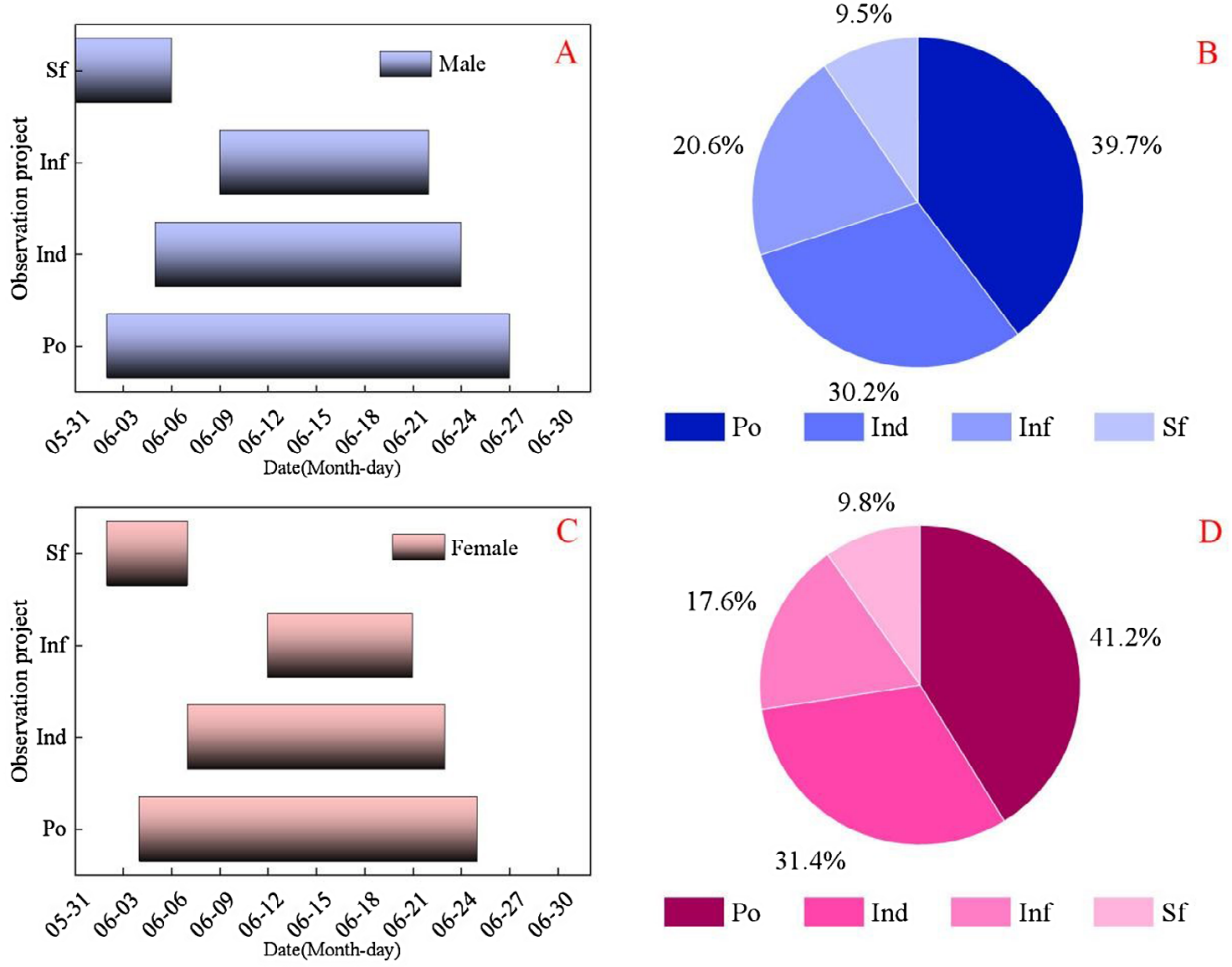
Phenological characteristics of female and male flowers of *Actinidia arguta* . (A) Flowering phenology of male; (B) Proportion of flowering period of male; (C) Flowering phenology of female; (D) Proportion of flowering period of female. Ind, individual; Inf, inflorescence; Po, population; Sf, single flower.

The flowering periods for female and male of *A. arguta* populations were 2nd June to 27th June and 4th June to 25th June, respectively. The peak flowering period for both was June 16th, and the durations of the flowering periods were 21 ± 2 and 25 ± 2 days, respectively (Figure 3A,C), accounting for 41.2% and 39.7% of the total flowering duration across the four levels (Figure 3B,D). The individual level flowering duration was 16 ± 2 and 19 ± 2 days, respectively, accounting for 31.4% and 30.2% of the flowering duration proportion across the four levels. The inflorescence level flowering duration was 9 ± 2 and 13 ± 2 days, respectively, accounting for 17.6% and 20.6% of the flowering duration proportion across the four levels. The individual flowering periods were 4 ± 1 and 3 ± 1 days, respectively, accounting for 9.8% and 9.5% of the flowering duration proportion across the four levels. The number of flowers per individual plant was 120 ± 25 and 103 ± 19, respectively, while the number of flowers per inflorescence was 9 ± 5 and 4 ± 4, respectively.

#### 3.1.2 Flowering Amplitude

**FIGURE 4:**
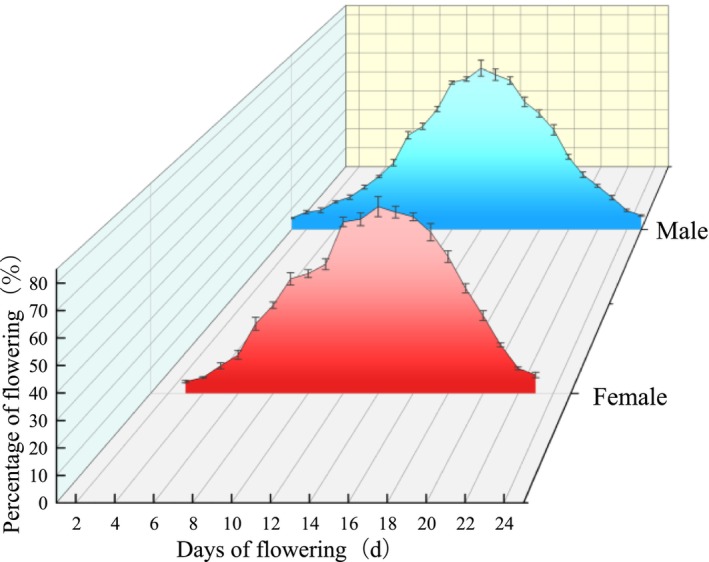
Flowering amplitude curves for female and male at the individual level of *Actinidia arguta*.

The flowering phenological progression curves for both female and male single *A. arguta* showed a single peak pattern. The proportion of flowers gradually increased with the flowering process; the female flowers reached the peak of 75.6% ± 2.1% after 11 days of flowering, the male flowers reached the peak of 78.5% ± 1.9% after 14 days of flowering, followed by a decline (Figure 4). The flowering synchrony indices at the individual level for female and male were 0.752 and 0.763, respectively.

### 3.2 Floral Characteristics

**FIGURE 5:**
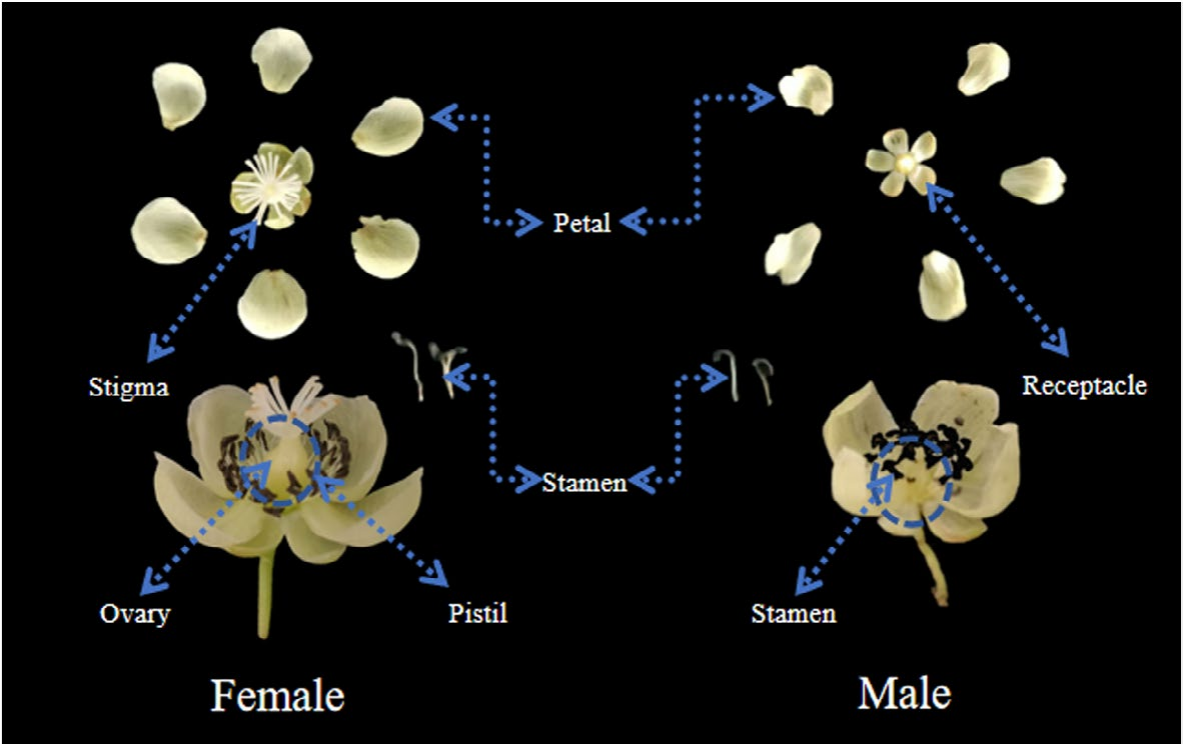
Comparison of morphological structure of female and male flowers of *Actinidia arguta*.

The female and male of the *A. arguta* are both white, crateriform. The dark colored structures surrounding the ovary in the female flower are sterile staminodes. The female flowers possess multi‐lobed stigmas arranged radially. The number of lobes is usually 15–25, filiform and mucilaginous. The stigmas are white and expanded when unpollinated, and contract and turn light yellow or brown after pollination. The male anthers are black, clustered, with approximately 25–45 anthers per flower (Figure 5).

**FIGURE 6:**
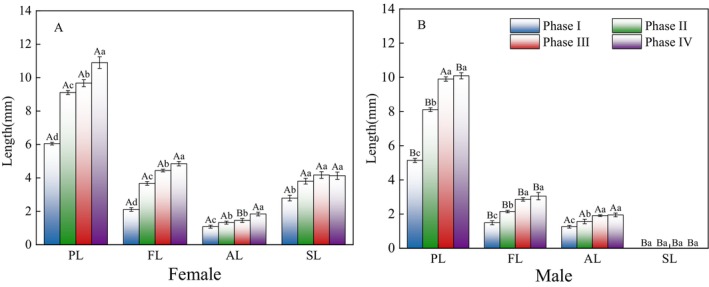
Comparison of corolla, filament, anther, and stigma lengths between female and male during the flowering of the *Actinidia arguta* . Different uppercase letters indicate significant differences in corolla, filament, anther, and stigma lengths between female and male at the same growth stage (*p* < 0.05); Different lowercase letters indicate significant differences in corolla, filament, anther, and stigma lengths of female or male at different growth stages (*p* < 0.05). AL, anther length; FL, filament length; PL, petal length; SL, stigma length.

Significant differences were observed in the corolla length of female and male *A. arguta* during Phase I, Phase II, and Phase IV (*p* < 0.05). Significant differences in filament length were observed in all four flowering phases (*p* < 0.05), and significant differences in anther length were found during Phase III (*p* < 0.05) (Figure 6).

### 3.3 Pollen Viability

**FIGURE 7:**
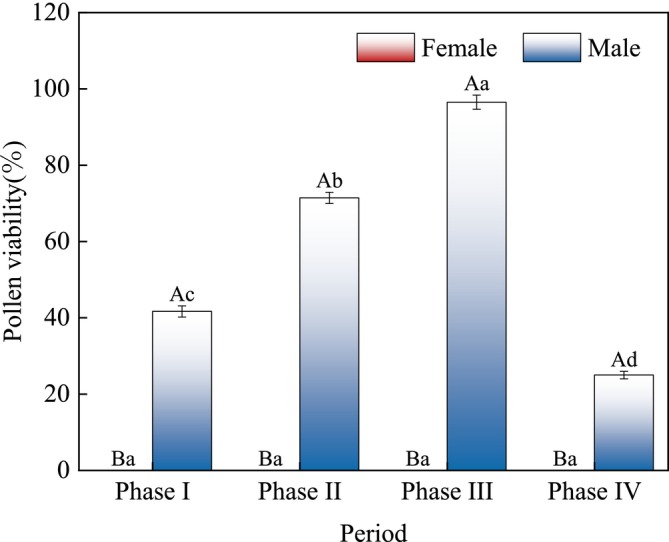
TTC staining method for detecting pollen viability in female and male of *Actinidia arguta* during four flowering stages. Different uppercase letters indicate significant differences in pollen viability between different types of flowers at the same stage (*p* < 0.05); Different lowercase letters indicate significant differences in pollen viability of the same type of flower at different stages (*p* < 0.05).

The pollen viability of male in *A. arguta* was 41.67% ± 1.48% at Phase I and was higher at 71.42% ± 1.46% during Phase II. Pollen viability reached its peak at 96.5% ± 1.86% during Phase III and was lowest at 22.5% ± 0.98% during Phase IV. During flowering, the pollen of female flowers showed no viability (Figure 7). The model margin of fit marginal *R* ^2^ = 0.84, indicating excellent fit. The results showed that sex had a highly significant positive effect on pollen viability (*F*_1,78_ = 189.62, *p* < 0.01, Est = 58.02 ± 1.86); the four periods also had a highly significant effect on pollen viability (*F*_3,78_ = 216.35, *p* < 0.01, Est = 43.21 ± 1.28).

Shapiro–Wilk test indicated that the pollen viability data followed a normal distribution (*W* = 0.95, *p* > 0.05); Levene test confirmed the data met the homogeneity of variance (*F* = 1.07, df = 378, *p* > 0.05). ANOVA results showed highly significant differences in pollen viability between sexes (*F*_1,78_ = 189.62, *p* < 0.01) and between the four periods (*F*_3,78_ = 216.35, *p* < 0.01). The reported effect size *η* ^2^ = 0.72 demonstrates that both gender and four periods exert significant impacts on pollen viability.

### 3.4 Stigma Receptivity

The stigma of the female of *A. arguta* began to be receptive when the flowers were unopened, but was the weakest at this time. The receptivity is relatively high during the early flowering, with the highest number of bubbles during the peak flowering, when the stigma is most receptive. Once all petals have fallen, the stigma becomes weak receptive (Table 1).

**TABLE 1:** Detection of stigma receptivity of *Actinidia arguta* female during the flowering.

Flowering phase	Phase I	Phase II	Phase III	Phase IV
Stigma receptivity	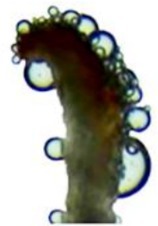	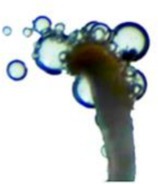	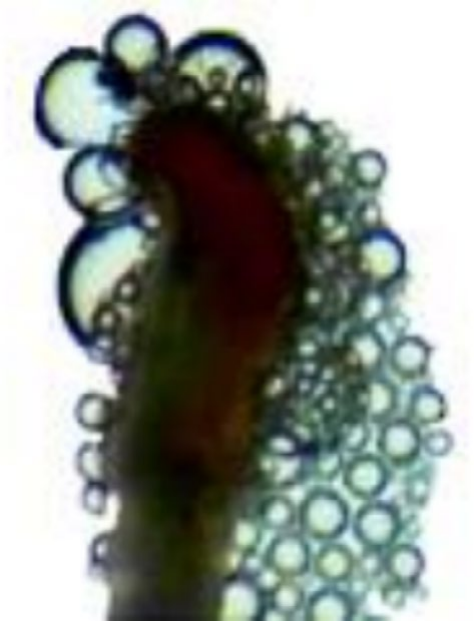	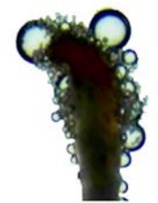

*Note:* Strength of stigma receptivity: Strongest (+++); Strong (++); Weak (+); Weakest (+/−).

### 3.5 Nectar Secretion of Female and Male

**FIGURE 8:**
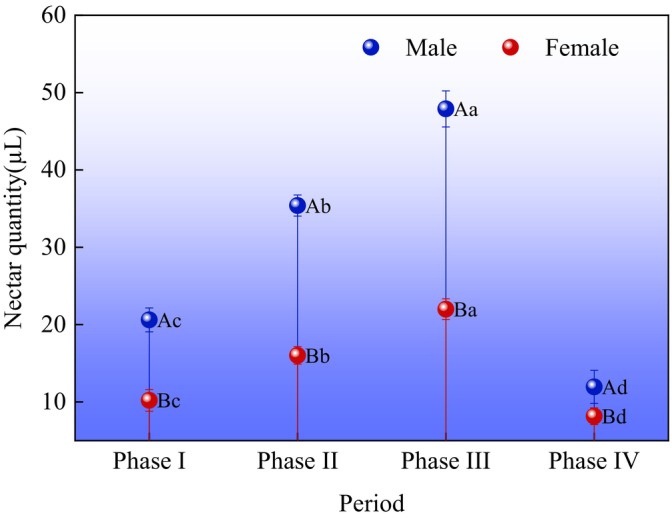
Nectar secretion in female and male of *Actinidia arguta* at four stages. Different uppercase letters indicate significant differences in nectar secretion between different types of flowers at the same stage (*p* < 0.05); Different lowercase letters indicate significant differences in nectar secretion of the same type of flower at different stages (*p* < 0.05).

The nectaries of both female and male *A. arguta* are located in the basal receptacle. Nectar secretion was the highest at Phase III, reaching 22 ± 1.33 and 47.9 ± 2.33 μL, respectively. Nectar secretion was the lowest at Phase IV, with 8.16 ± 1.08 and 11.96 ± 2.14 μL, respectively (Figure 8). The model margin of fit marginal *R* ^2^ = 0.82. Results demonstrated that sex had a highly significant positive effect on nectar secretion (*F*_1,78_ = 42.86, *p* < 0.01, Est = 14.87 ± 1.21), with male flowers exhibiting significantly higher nectar secretion than females. The four periods also showed a highly significant effect on nectar secretion (*F*_3,78_ = 58.37, *p* < 0.01, Est = 18.33 ± 1.05).

Shapiro–Wilk test confirmed normal distribution of nectar secretion data (*W* = 0.96, *p* > 0.05). Levene test verified homogeneity of variance (*F* = 1.12, df = 3,78, *p* > 0.05). ANOVA revealed highly significant differences in nectar secretion between sexes (*F*_1,78_ = 42.86, *p* < 0.01) and among the four periods (*F*_3,78_ = 58.37, *p* < 0.01). The reported effect size *η* ^2^ = 0.68 demonstrates that both sex and four periods exert substantial influence on nectar secretion.

### 3.6 Morphological Observations of Anthers, Pollen Grains, and Stigmas

**FIGURE 9:**
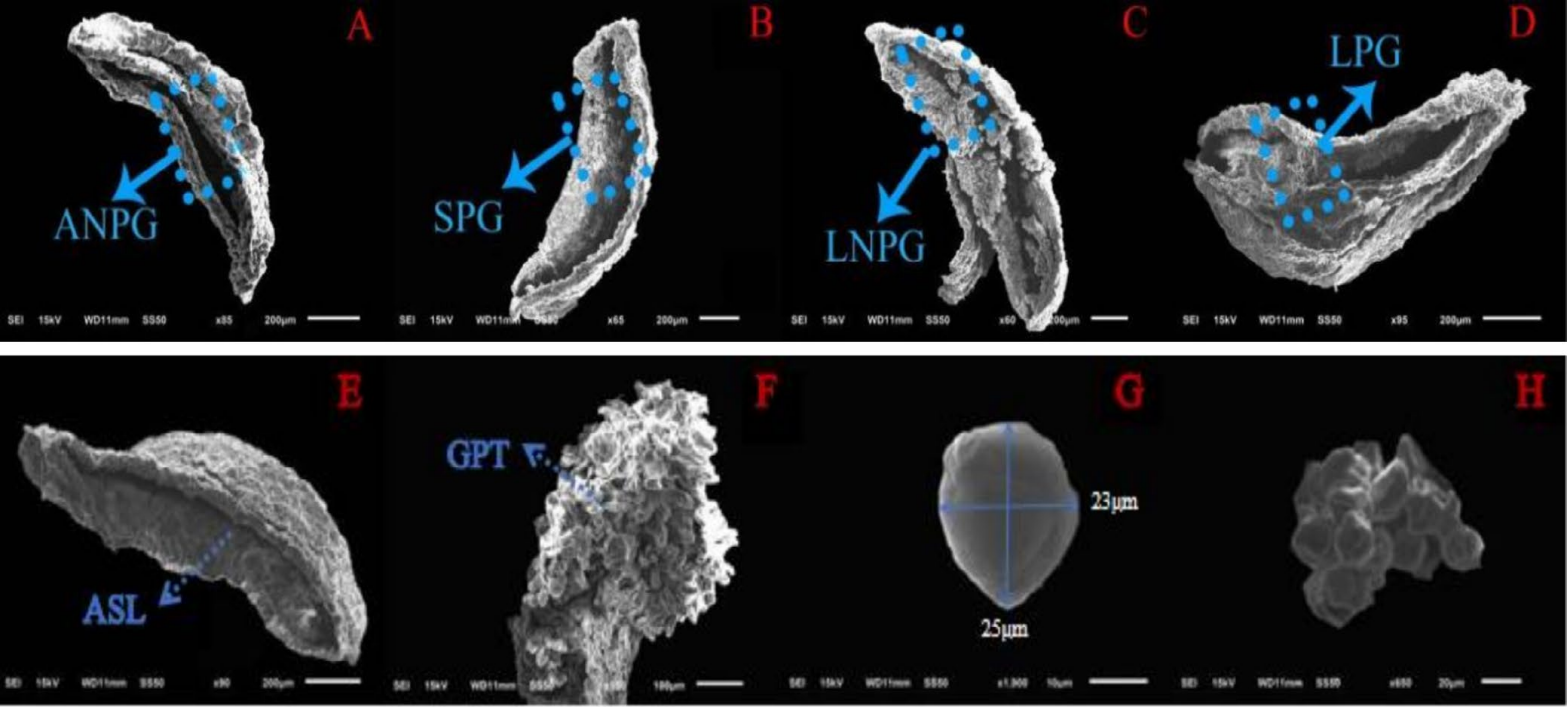
Morphology of anthers, pollen grains, and stigma in *Actinidia arguta* . (A–D) Stage of anther dehiscence in male; (E) incomplete anther dehiscence in female; (F) pollen tubes germinated on the stigma surface; (G) pollen grain; (H) pollen grain aggregation. ANPG, almost no pollen grains released from the anther; ASL, anthers split longitudinally; GPT, germination of pollen tubes; LNPG, large number of pollen grains released from the anther; LPG, a few pollen grains released from the anther; SPG, some pollen grains released from the anther.

Observations indicate that the anthers of *A. arguta* are long elliptical or sagittate, bilaterally symmetric, and dehisce longitudinally. Pollen is gradually released during the flowering of single flowers. Directly measured data indicate that the anther diameter from Phases I to IV were 116 ± 8, 322 ± 22, 395 ± 25, and 136 ± 14 μm, respectively (Figure 9A–D). Observations indicate that there are a large number of germinated pollen tubes on the stigma surface (Figure 9F). The pollen is small, nearly spherical to long spherical, with 3 grooves and groove length, and the terminal end is more pointed with moderate opening. Directly measured data indicate that the polar measures 21.2 ± 1.9 μm, the equatorial 15.3 ± 1.5 μm, with a P/E ratio of 1.00–1.15. The outer wall is thin and finely granular (Figure 9G).

**FIGURE 10:**
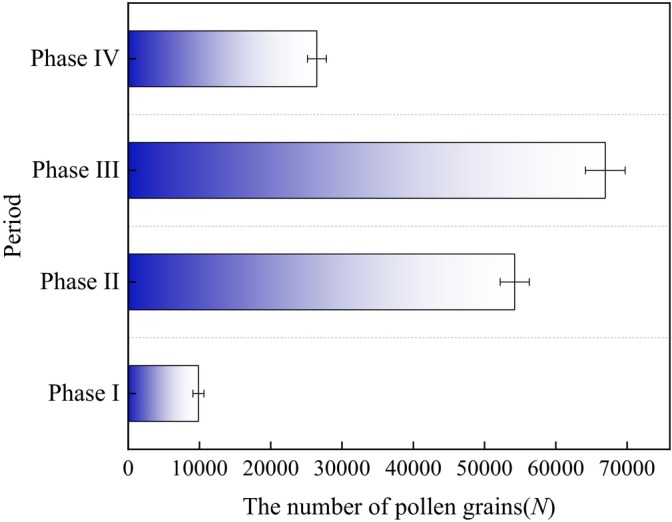
Process of pollen grain release in male flowers of *Actinidia arguta*.

**FIGURE 11:**
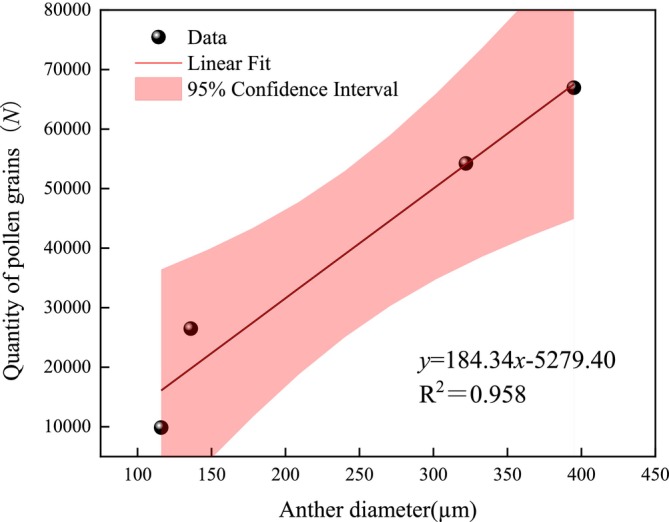
Linear regression analysis of the anther diameter and the number of pollen grains in a single flower of *Actinidia arguta* . *x*, the anther diameter; *y*, the number of pollen grains in a single flower, regression equation: *y* = 184.34*x* − 5279.40, *R* ^2^ = 0.958, *p* < 0.01.

During the Phase I, the anthers of male showed the smallest aperture, releasing a mean of 9860 ± 767 pollen grains. Gradually increasing during the Phase II, the quantity of pollen grains released was 54,230 ± 2036 grains. At the Phase III, the anthers opened to their maximum extent, releasing a mean of 66,945 ± 2790 pollen grains. The number of pollen grains released decreased toward the Phase IV, amounting to 26,475 ± 1322 grains (Figure 10). Correlation analysis reveals significant relationship between anther diameter and pollen grains release number (*p* < 0.01, *r* = 0.976 > 0), demonstrating a strong positive correlation between the two variables. The correlation analysis of the anther diameter and the number of pollen grains single flower of the male flowers showed that the anther diameter and the number of pollen grains single flower had a very significant linear positive correlation, and the linear regression equation was: *y* = 184.34*x* − 5279.40. The high goodness of fit (*R* ^2^ = 0.958) indicates that the regression equation is highly significant (*p* < 0.01). Within the study observation range, the number of pollen grains single flower increases significantly with the increase in anther diameter (Figure 11).

### 3.7 Pollination Characteristics

#### 3.7.1 Insect Visiting Behavior and Identification of Pollinator Species

**FIGURE 12:**
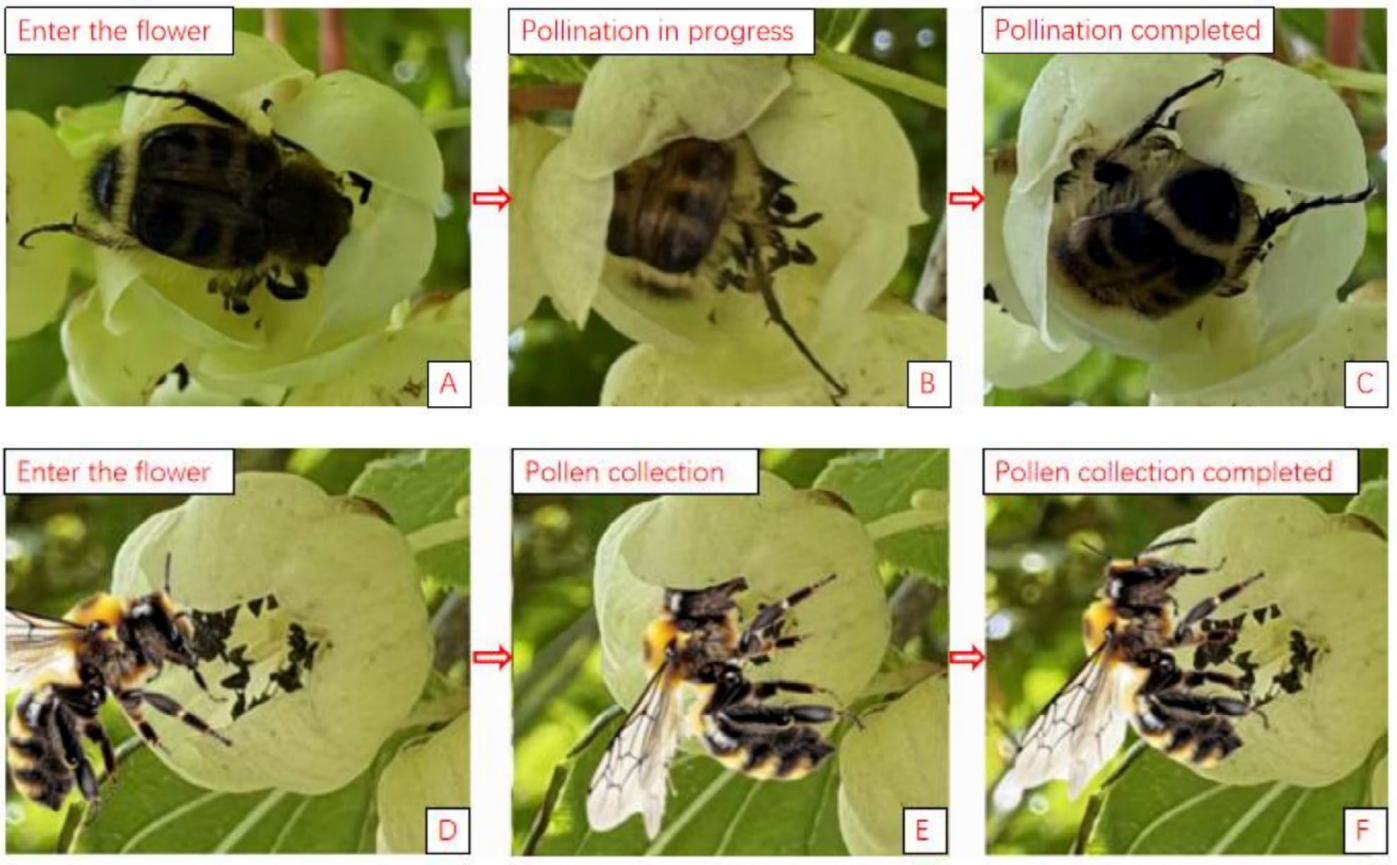
Visiting process of primary pollinator insects of *Actinidia arguta*. (A–C) Visiting process to female of *Bombus*; (D–F) visiting process to male of *Apis cerana*.

**FIGURE 13:**
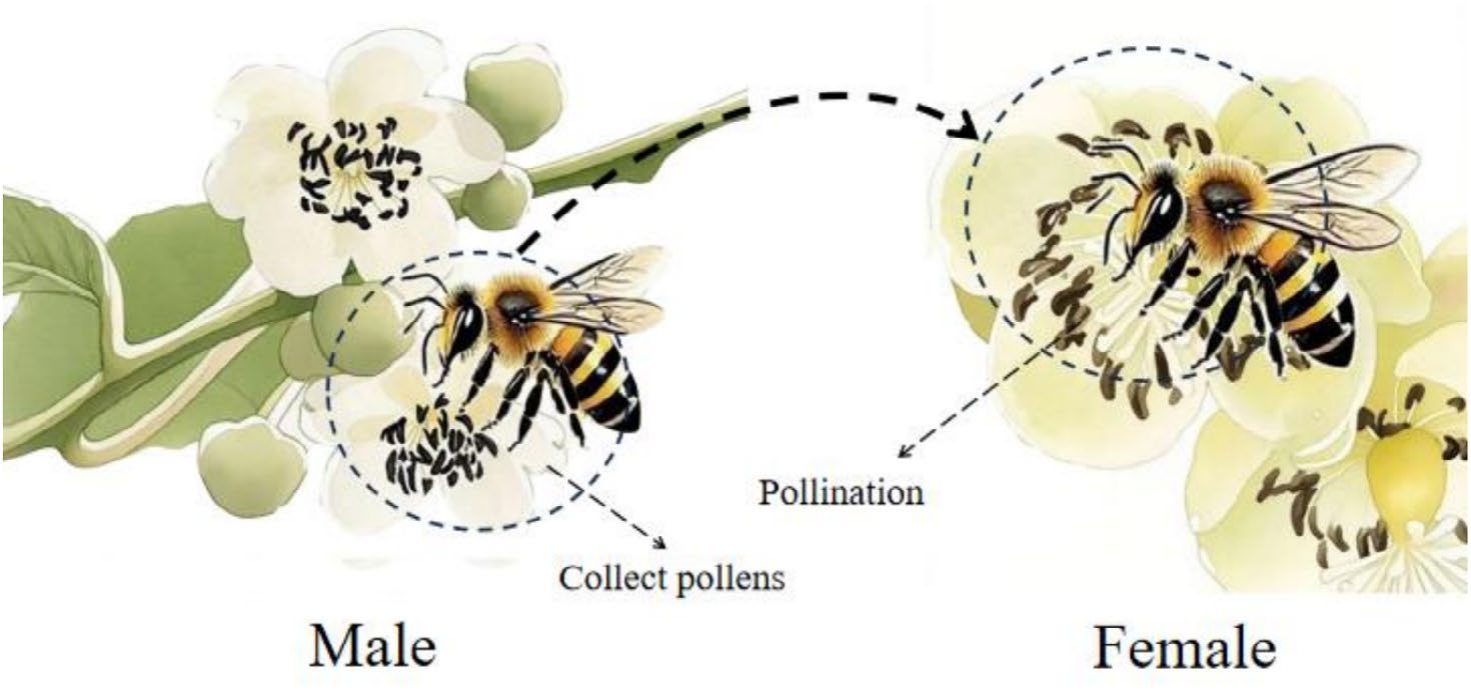
Schematic diagram of pollinator insects visiting *Actinidia arguta*.

*Apis cerana* and *Bombus* are the effective pollinator insects of *A. arguta* . Before visit, pollinator insects first survey their surroundings, observing the blooms row by row along the middle and upper sections of *A. arguta* . Then they fly toward the visited flowers. When pollinator insects visit, they alight directly or crawl onto the stamens of the male flowers, flapping their front legs back and forth to collect pollen in the pollen baskets on their hind legs. After completing pollen collection from one flower, the insects fly to the inflorescence and nearby flowers on the plant, repeating this collecting pollen process. They then proceed to female flowers. Moving across the female, the pollen adhering to its body and that carried on its hind legs comes into contact with the stigma, completing pollination (Figures 12 and 13).

#### 3.7.2 Visiting Frequency

**FIGURE 14:**
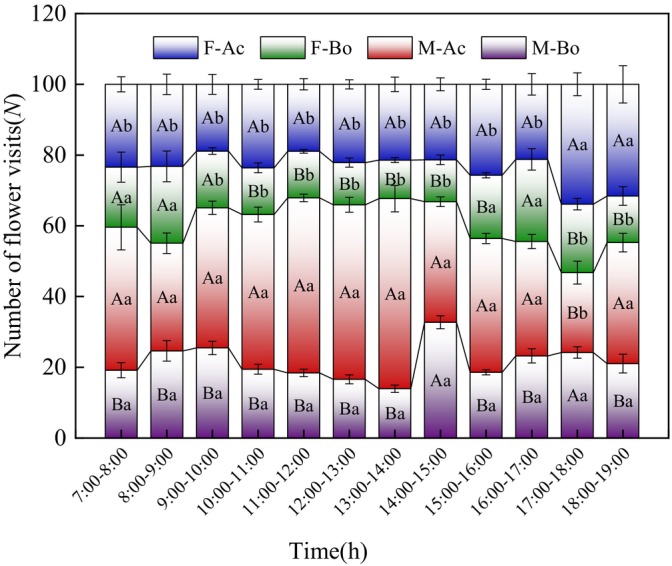
Visiting frequency of primary pollinator insects of *Actinidia arguta*. F‐Ac, female‐ *Apis cerana* ; F‐Bo, female‐*Bombus*; M‐Ac, male‐ *A. cerana* ; M‐Bo, male‐*Bombus*. Different uppercase letters indicate significant differences in visiting frequencies between different pollinator insects on female and male (*p* < 0.05); Different lowercase letters indicate significant differences in visiting frequencies of the same pollinator on female and male (*p* < 0.05).

The staying time of *A cerana* on a single inflorescence of female and male was 95 ± 8 and 120 ± 10 s, respectively, and the staying time on a single flower was 35 ± 5 and 42 ± 4 s, respectively. The staying time of *Bombus* on a single inflorescence of female and male was 64 ± 4 and 59 ± 6 s, respectively, and the staying time on a single flower was 21 ± 2 and 28 ± 3 s, respectively. The visiting frequency of *A. cerana* on both female and male was the highest between 13:00 and 14:00, with the maximum number of visits being 63 ± 6 and 158 ± 11, respectively. The peak visiting time of *Bombus* on male was between 14:00 and 15:00, with the mean number of visiting being 72 ± 4, and the peak flower‐visiting time on female flowers was between 13:00 and 14:00, with the mean number of visiting being 32 ± 2 (Figure 14). The visiting frequency of *A. cerana* was higher than that of *Bombus*. There were significant differences in the visiting frequency of *A. cerana* and *Bombus* on male flowers between 7:00–14:00, 15:00–17:00, and 17:00–19:00 (*p* < 0.05), and significant differences in their visiting frequency on female between 10:00–16:00 and 17:00–19:00 (*p* < 0.05).

#### 3.7.3 Pollination Efficiency

The number of pollen grains carried away by *A. cerana* and *Bombus* after a single visit to male was 21,007 ± 1232 and 15,520 ± 822, respectively. Comparative validation showed no significant difference in pollen loss (*t* = 0.38, *p* = 0.706 > 0.05). The number of pollen grains deposited on the stigma after a single visit was 2566 ± 119 and 1527 ± 102, respectively. Their pollination efficiencies were 12.21% ± 0.13% and 9.88% ± 1.16%, respectively, with a significant difference between the two (*p* < 0.05) (Table 2).

**TABLE 2:** Pollination efficiency of two visiting insects.

Pollinator	Number of pollen grains deposited on stigma after single visit (*N*)	Number of pollen grains carried away after single visit (*N*)	Pollination efficiency (%)
*Bombus*	1527 ± 102	15,520 ± 822	9.88 ± 1.16^b^
*Apis cerana*	2566 ± 119	21,007 ± 1232	12.21 ± 0.13^a^

*Note:* Different lowercase letters indicate significant differences in pollination efficiency between pollinator insects (*p* < 0.05).

### 3.8 Artificial Pollination Experiments

The fruit‐set rate of *A. arguta* under natural pollination was 56.10% ± 2.56%. Female treated with bagging only did not set fruit, indicating that there was no apomixis. The fruit set under supplementary pollination was highest at 87.21% ± 5.09% and significantly higher than natural pollination (*p* < 0.05), indicating that *A. arguta* sexual reproduction is highly dependent on pollinator insects (Table 3).

**TABLE 3:** Supplementary pollination experiment of *Actinidia arguta* .

Treatment	Number of treated flowers (*N*)	Number of flowers producing fruit (*N*)	Fruit‐set rate (%)
Natural pollination	60	34	56.10 ± 2.56^b^
Supplementary pollination	60	52	87.21 ± 5.09^a^
Bagging	60	0	0^c^

*Note:* Different letters indicate significant differences in fruit‐set rate under different treatments (*p* < 0.05).

## 4 Discussion

### 4.1 Interaction Between Anther Dehiscence Mode and Sequential Pollen Presentation in *A. arguta*

Anther dehiscence modes include longitudinal, poricidal, valvate, and transverse dehiscence, which are mutually adaptive to pollination strategies (Ma et al. 2024). As the final step in the anther development stage, anther dehiscence is crucial for the release of mature pollen grains, pollination and fertilization (Thangasamy et al. 2011). Gradual anther dehiscence, which sequentially presents pollen to pollinating insects, is one of the typical floral characteristics of plants to improve male fitness (Ma 2011; Wu et al. 2025). The interaction between the process of sequential pollen presentation and pollinators enables the accurate and efficient transfer of pollen to the stigmas of other flowers, thereby enhancing male fitness and ensuring the success of cross‐pollination (Yang 2016). In environments with a lack of pollinators, *Gagea nigra* extends its pollen release period through sequential pollen presentation, improves pollen transfer efficiency, and enhances the plant's ability to adapt to environmental changes to ensure successful reproduction (Zheng et al. 2016). Pollen release requires a high degree of temporal coordination among multiple metabolic processes, so that pollen dispersal occurs at the optimal time to maximize the efficiency of self‐pollination and cross‐pollination (Hua 2017). The results of this study showed that the anther dehiscence mode of *A. arguta* is longitudinal dehiscence, with “unzipping” pollen dispersal. There is a positive correlation between the size of the anther opening and the amount of pollen presented. Compared to *A. chinensis* and *A. deliciosa* , the pollen of *A. arguta* is smaller in volume and exhibits more delicate ornamentation (Qi et al. 2017). The pollen presentation strategy is sequential presentation, which controls the number of pollen grains presented to pollinators by the anther at one time. By presenting pollen to pollinating insects in small, multiple batches, the loss during pollen presentation and transfer is reduced, and the proportion of pollen deposited on the stigma is increased, thereby improving male fitness.

### 4.2 Mutual Adaptation Between Nectar Secretion Strategies of Female and Male *A. arguta* and Pollination

Nectar is the most effective reward for attracting and influencing pollinator behavior, and it is also a universal “currency” used by most flowering plants to attract pollinators (Nicolson and Thornburg 2007). The secretion quantity and secretion pattern of plant nectar have evolved synergistically over time to align with pollinators' visitation behaviors, forming a bidirectional adaptive and matching relationship (Zhao 2017). The secretion of nectar can be used as a supplementary strategy for the utilization of pollen resources. The secretion characteristics of nectar can affect the stay time of pollinating insects on a single flower, the removal amount of pollen grains, and the deposition amount of pollen grains on stigma (Huang and Guo 2000). It is closely related to the foraging behavior of pollinating insects and the reproductive capacity of individual flowers (Zhang et al. 2011). Plants balance “pollen limitation” and “reward cost” by regulating nectar secretion. A higher nectar volume can attract more pollinators and prolong their visiting time, thereby improving pollen transfer efficiency (Souza et al. 2017; Cnaani et al. 2006). Male secrete abundant nectar as a generous reward to attract more visitors, thereby enhancing pollen transport efficiency; female secrete less nectar because they allocate more resources to fruit and seed development (Briones et al. 2019). This study revealed that males *A. arguta* exhibit significantly higher nectar secretion than females, with peak secretion occurring during the peak flowering period. This secretion pattern attracts pollinating insects and prolongs their time on male plants, thereby increasing the pollen carried by pollinators. Additionally, the nectar secretion period effectively overlaps with the pollen viability and stigma receptivity phases of different flowering stages, which not only reduces pollen waste but also promotes effective cross‐pollination.

### 4.3 Interaction Between Sexual Dimorphism and Reproduction in Dioecious Plants

Sexual dimorphism in dioecious plants refers to a series of systematic differences exhibited between different sexes (male and female) (Wang et al. 2025). The evolution of dimorphism is reflected in differences in sexual reproductive traits such as flowering phenology, floral characteristics, and floral rewards (Shu et al. 2024). For *Arisaema heterophyllum* Blume, sexual dimorphism is mainly manifested in flowering time and reproductive structures (Kinoshita 1986). The results of this study are consistent with this finding. Female *A. arguta* flowers do not produce pollen, and male flowers lack stigmas. Male start flowering earlier than female. The early flowering of males ensures that there is sufficient pollen available for fertilization and fruit set when females bloom, increasing the probability of ovule fertilization within the same species and thus achieving higher reproductive fitness. Dimorphism is also reflected in life‐history strategies and resource allocation: males usually invest more in pollen dispersal (producing more small flowers and having a longer flowering period) and improve reproductive success by increasing pollen production, while females undertake the reproductive growth of seeds and fruits (Bullock and Bawa 1981; Zeng et al. 2022). In this study, the floral size of females of *A. arguta* was significantly larger than males, and the larger flower size could serve as a visual signal to attract pollinators. The visiting frequency of males is significantly higher than that of females; males attract visiting insects through the number of flowers and pollen grains, which is beneficial to entomophily, thereby improving the fruit set rate and achieving the optimal resource allocation.

## 5 Conclusions

*Actinidia arguta* adopts a sequential pollen presentation mechanism for anther pollen dispersal. Through longitudinal anther dehiscence, it improves male fitness, enhances pollination efficiency, and promotes cross‐pollination. *A. arguta* exhibits sexual dimorphism and is a typical entomophilous plant, relying on pollinating insects to transfer pollen from male to the stigmas of female to complete pollination and subsequent fruit set. The visiting frequency of male is significantly higher than female; male attract pollinators to collect pollen through the number of flowers and pollen grains. Male secrete a large amount of nectar, while female secrete less nectar; this nectar secretion pattern attracts pollinating insects (including *A. cerana* and *Bombus*) to visit, increasing the duration pollinators spend on individual flowers leads which to an increase in the number of pollen grains transferred by pollinators. The dioecious plants have a reproductive structure that force the pollination by different flowers, which fundamentally avoids the self‐pollination decline and improves the genetic diversity and environmental adaptability of the offspring, and at the same time reduces the invalid loss of pollen, optimizes the utilization efficiency of pollen resources, and improves the pollination and reproduction efficiency, which is an important evolutionary choice of plants to adapt to the environment.

## Author Contributions

**Kexin Sun:** conceptualization (equal), data curation (equal), formal analysis (equal), investigation (equal), methodology (equal), writing – original draft (equal). **Xueying Yang:** data curation (equal), investigation (equal), resources (equal), software (equal). **Shanlin Yang:** conceptualization (equal), funding acquisition (equal), methodology (equal), project administration (equal), writing – review and editing (equal). **Rongrong Cui:** formal analysis (equal), investigation (equal), project administration (equal), supervision (equal). **Yan Ji:** data curation (equal), investigation (equal), visualization (equal). **Yuting Hu:** data curation (equal), investigation (equal), visualization (equal). **Renjie Li:** data curation (equal), investigation (equal). **Dingxu Jin:** supervision (equal), validation (equal).

## Conflicts of Interest

The authors declare no conflicts of interest.

## Supporting information


**Data S1:** ece373247‐sup‐0001‐supinfo.zip.

## Data Availability

The data supporting the findings of this study are included as Supporting Information with this manuscript and are available via the journal's website upon publication. All data supporting this study are include in the article and its Supporting Information.
