# Supplementary figures and images for: A New Insight Into Pollen Release and Presentation in Actinidiaceae Plants: The Case Study of Dioecious Actinidia arguta
Source: Ecol Evol. 2026 Apr 3;16(4):e73247. doi: 10.1002/ece3.73247 (PMC13052302; doi:10.1002/ece3.73247)

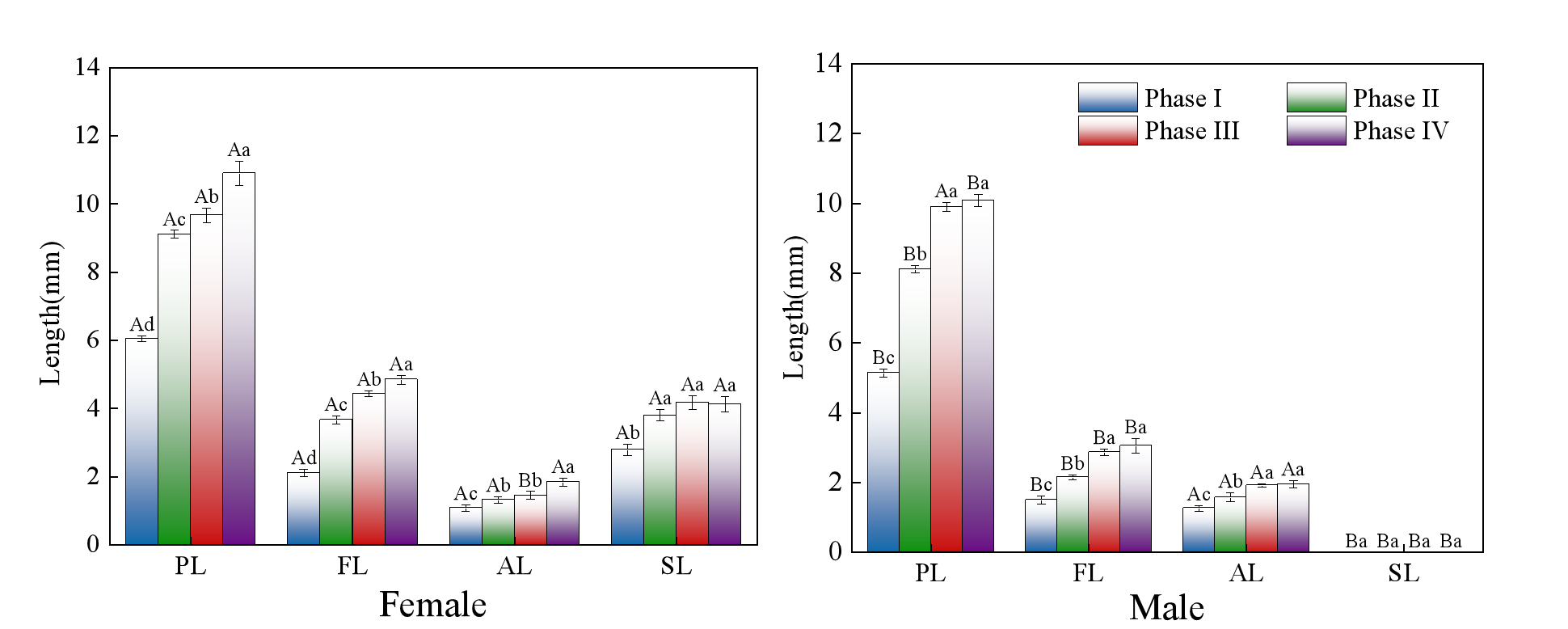

Supplement: Supplementary file 1 — Data S1: ece373247‐sup‐0001‐supinfo.zip. [file ECE3-16-e73247-s001.zip › supinfo file/Comparison of corolla, filament, anther, and stigma lengths between female and male.png]

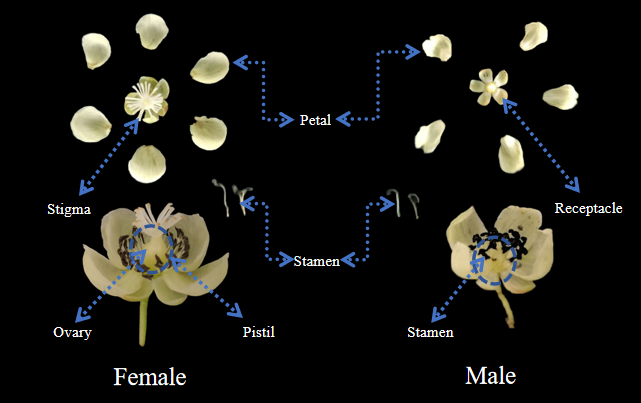

Supplement: Supplementary file 1 — Data S1: ece373247‐sup‐0001‐supinfo.zip. [file ECE3-16-e73247-s001.zip › supinfo file/Floral characteristics of female and male.png]

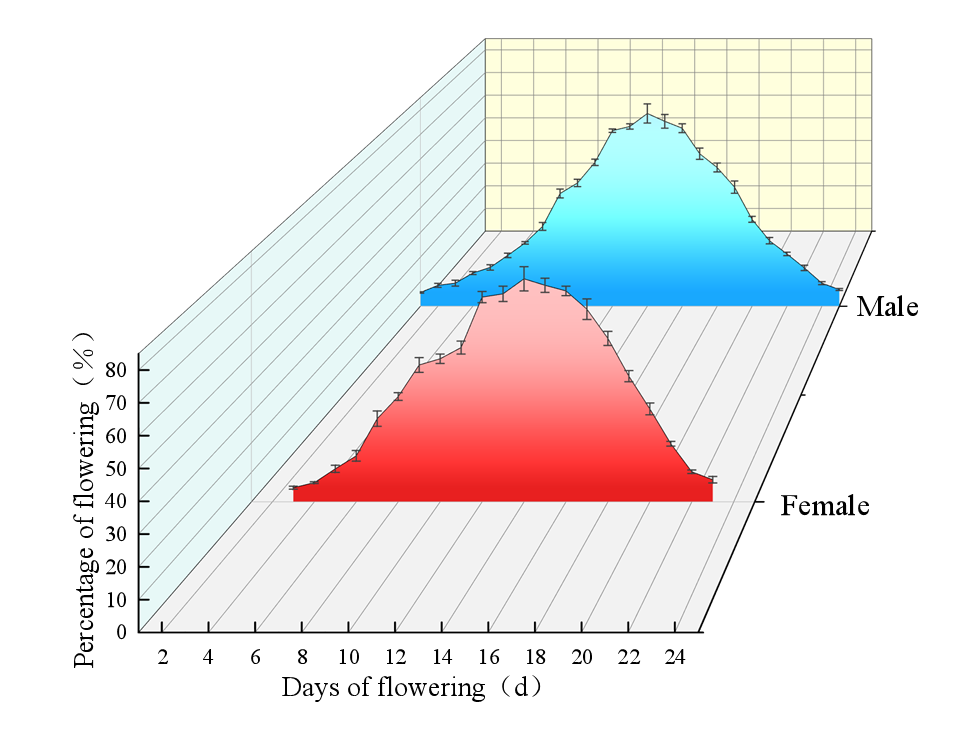

Supplement: Supplementary file 1 — Data S1: ece373247‐sup‐0001‐supinfo.zip. [file ECE3-16-e73247-s001.zip › supinfo file/Flowering amplitude curves for female and male.png]

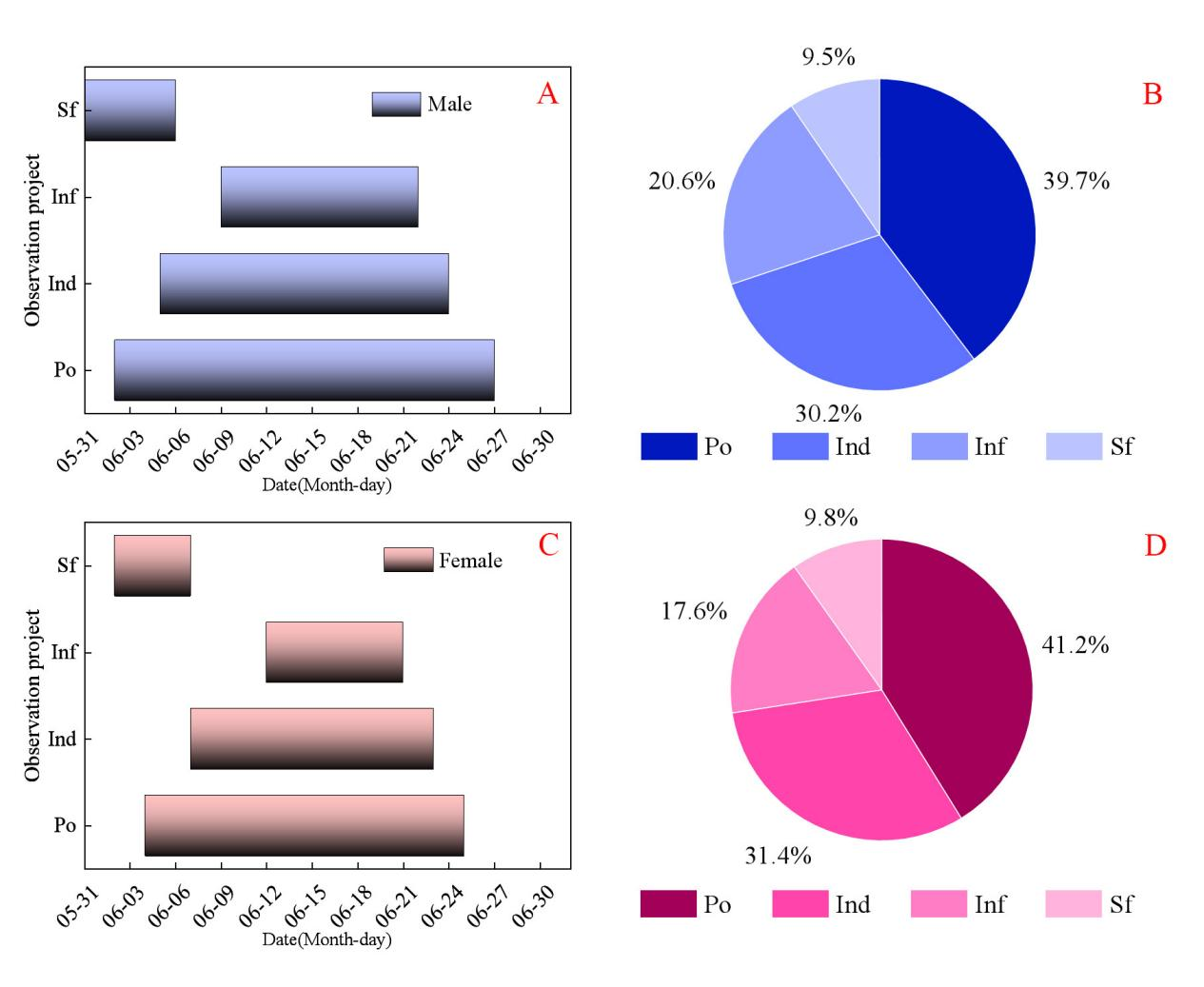

Supplement: Supplementary file 1 — Data S1: ece373247‐sup‐0001‐supinfo.zip. [file ECE3-16-e73247-s001.zip › supinfo file/Flowering phenology of female and male.png]

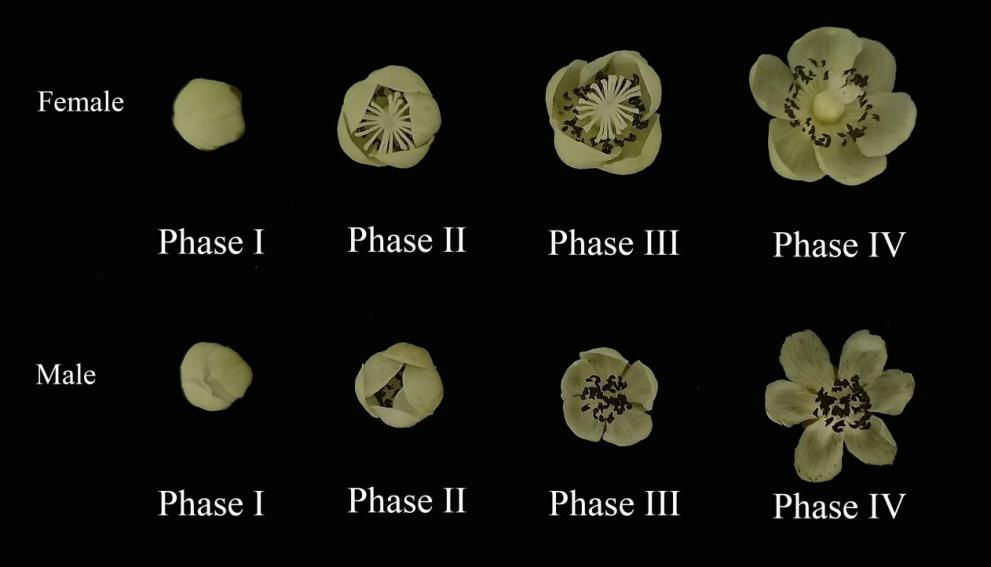

Supplement: Supplementary file 1 — Data S1: ece373247‐sup‐0001‐supinfo.zip. [file ECE3-16-e73247-s001.zip › supinfo file/Flowering progression of female and male.png]

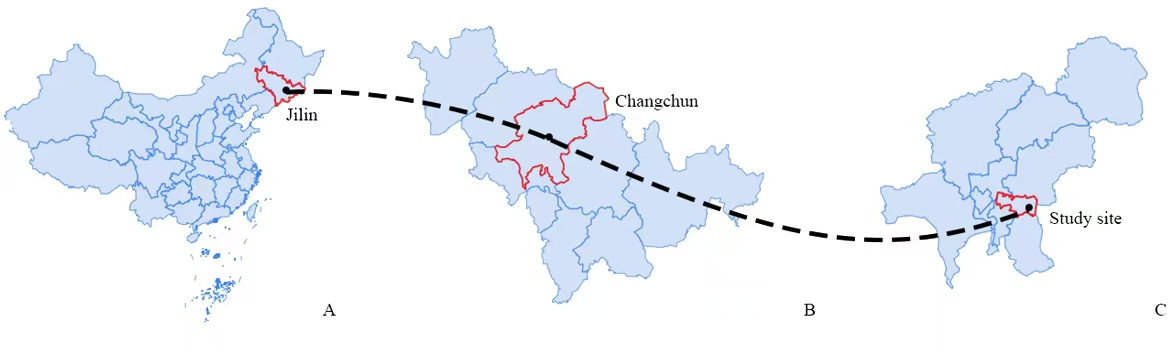

Supplement: Supplementary file 1 — Data S1: ece373247‐sup‐0001‐supinfo.zip. [file ECE3-16-e73247-s001.zip › supinfo file/Geographical location of the study area.png]

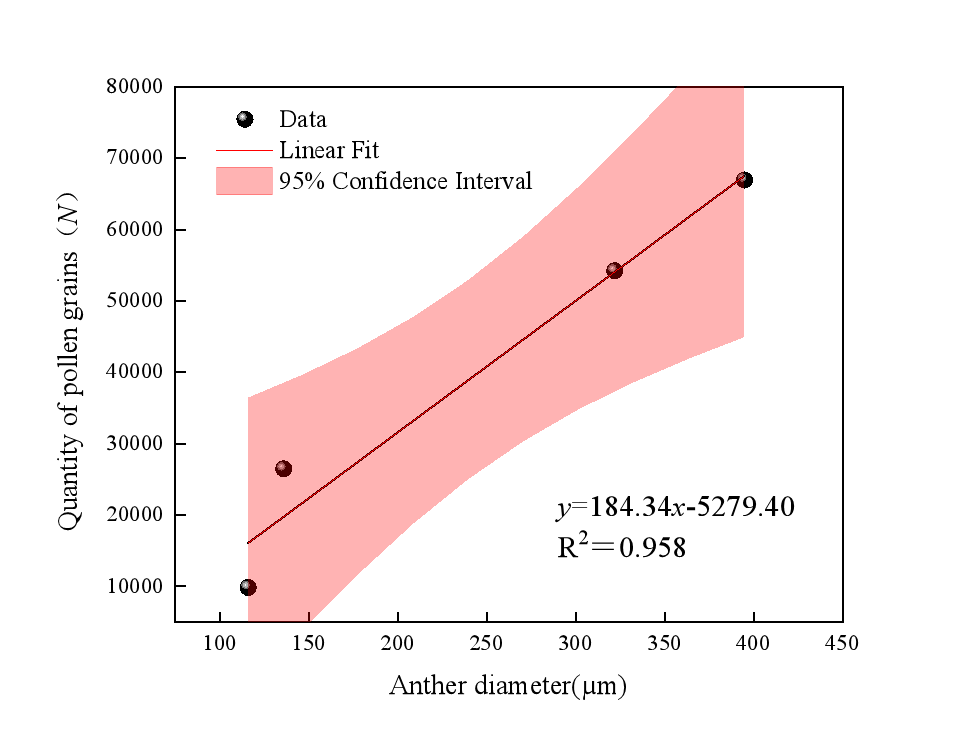

Supplement: Supplementary file 1 — Data S1: ece373247‐sup‐0001‐supinfo.zip. [file ECE3-16-e73247-s001.zip › supinfo file/Linear regression analysis of the anther diameter and the number of pollen grains.png]

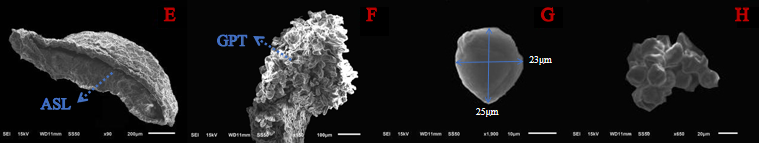

Supplement: Supplementary file 1 — Data S1: ece373247‐sup‐0001‐supinfo.zip. [file ECE3-16-e73247-s001.zip › supinfo file/Morphology of anthers, pollen grains, and stigma.png]

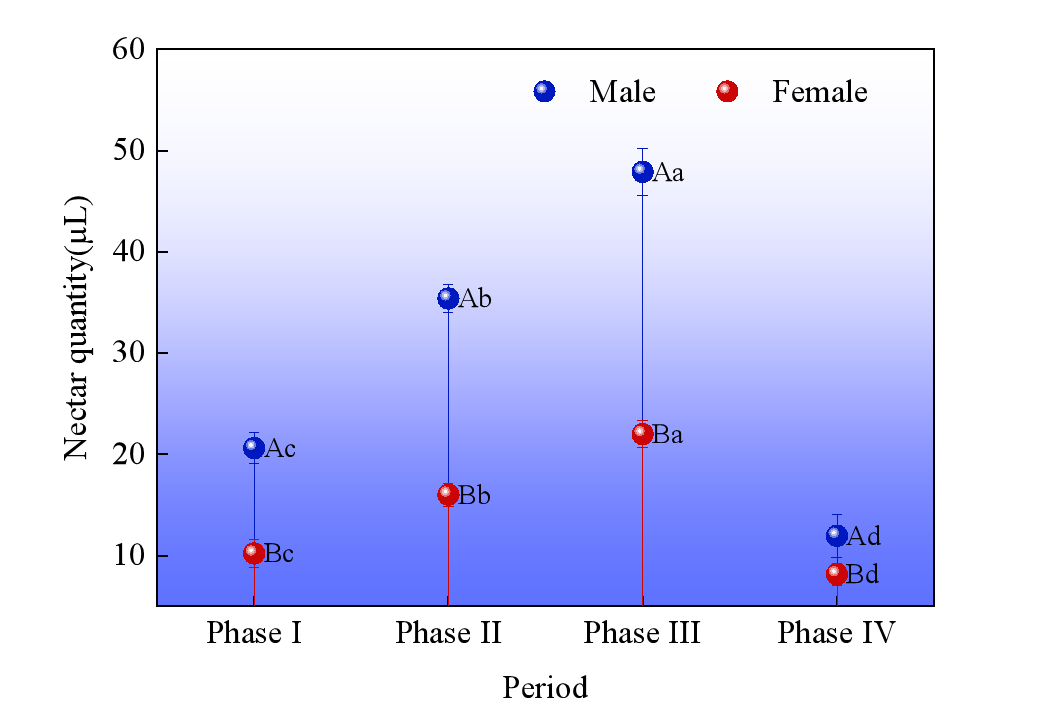

Supplement: Supplementary file 1 — Data S1: ece373247‐sup‐0001‐supinfo.zip. [file ECE3-16-e73247-s001.zip › supinfo file/Nectar secretion in female and male.png]

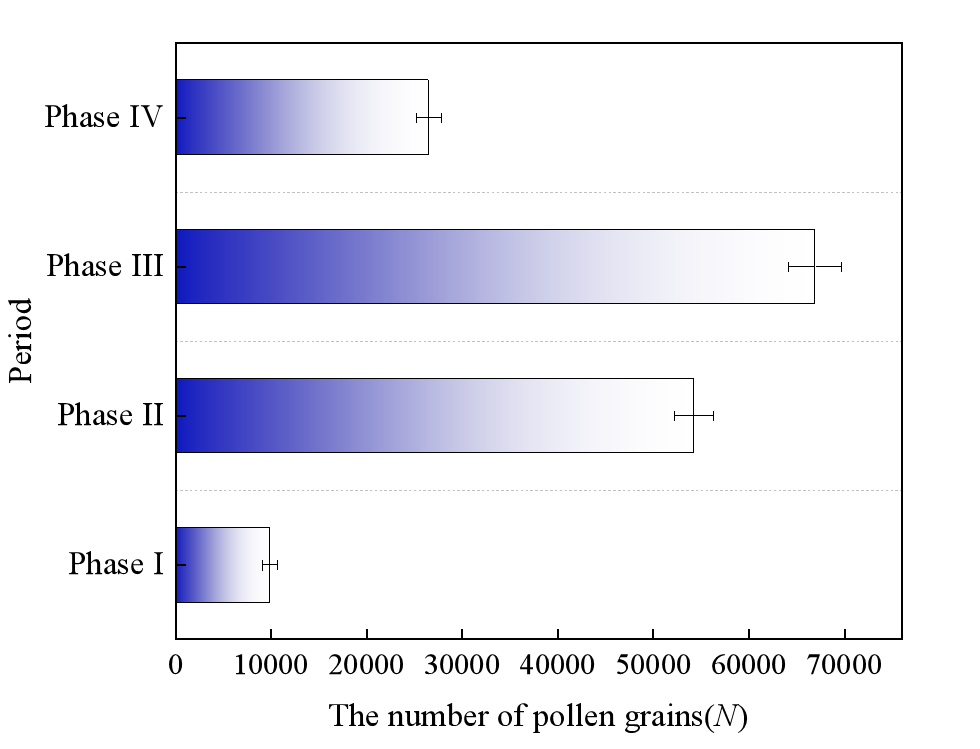

Supplement: Supplementary file 1 — Data S1: ece373247‐sup‐0001‐supinfo.zip. [file ECE3-16-e73247-s001.zip › supinfo file/Process of pollen grain release in male flowers.png]

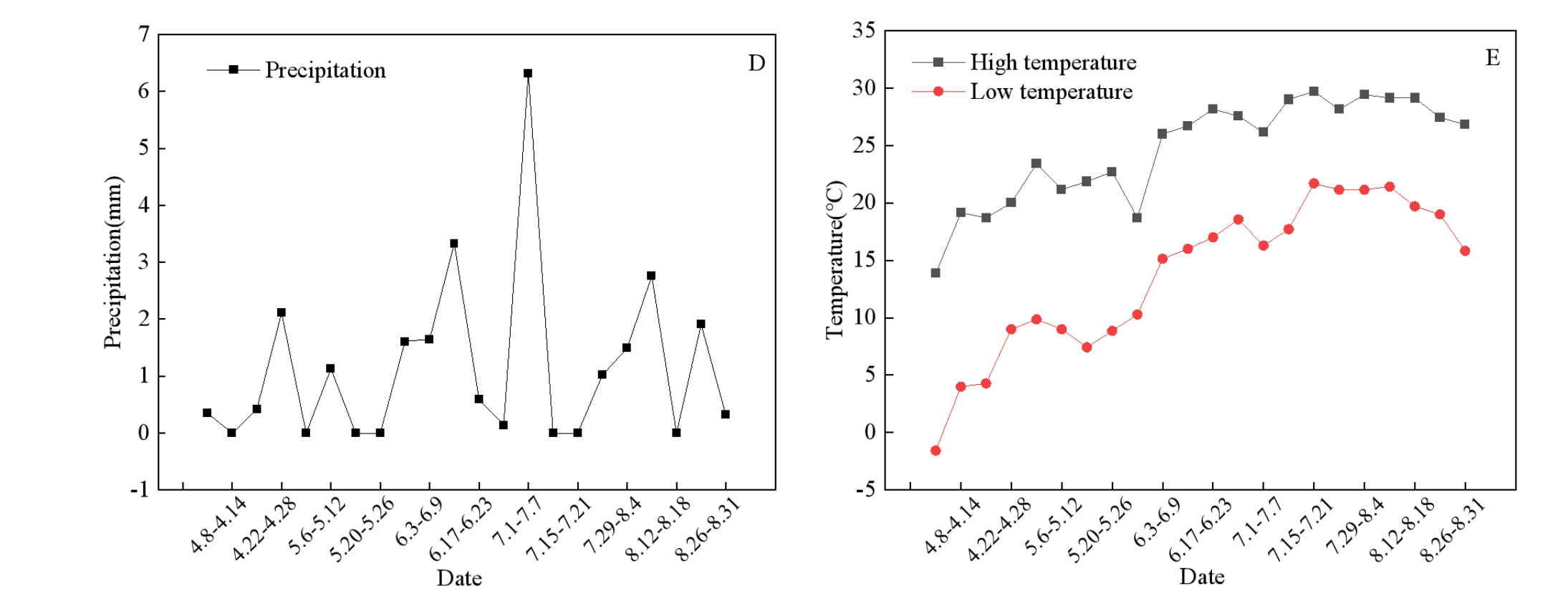

Supplement: Supplementary file 1 — Data S1: ece373247‐sup‐0001‐supinfo.zip. [file ECE3-16-e73247-s001.zip › supinfo file/rainfall during the flowering period.png]

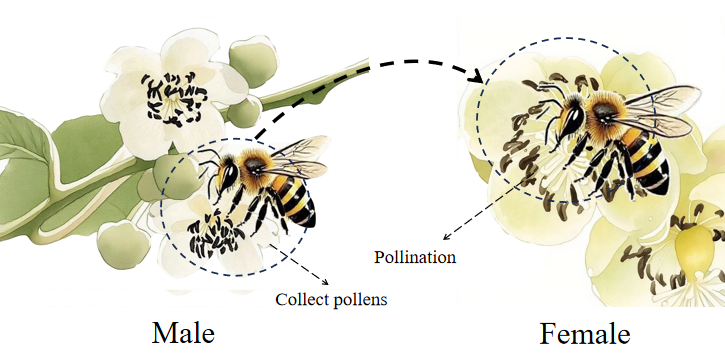

Supplement: Supplementary file 1 — Data S1: ece373247‐sup‐0001‐supinfo.zip. [file ECE3-16-e73247-s001.zip › supinfo file/Schematic diagram of pollinator insects visiting.png]

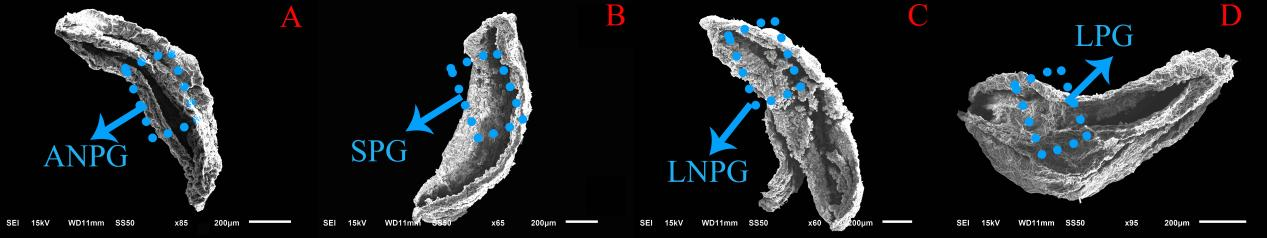

Supplement: Supplementary file 1 — Data S1: ece373247‐sup‐0001‐supinfo.zip. [file ECE3-16-e73247-s001.zip › supinfo file/Stage of anther dehiscence in male.png]

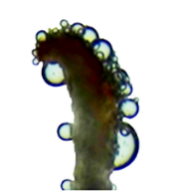

Supplement: Supplementary file 1 — Data S1: ece373247‐sup‐0001‐supinfo.zip. [file ECE3-16-e73247-s001.zip › supinfo file/stigma receptivity I.png]

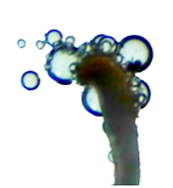

Supplement: Supplementary file 1 — Data S1: ece373247‐sup‐0001‐supinfo.zip. [file ECE3-16-e73247-s001.zip › supinfo file/stigma receptivity II.png]

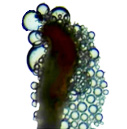

Supplement: Supplementary file 1 — Data S1: ece373247‐sup‐0001‐supinfo.zip. [file ECE3-16-e73247-s001.zip › supinfo file/stigma receptivity III.png]

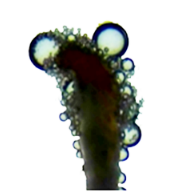

Supplement: Supplementary file 1 — Data S1: ece373247‐sup‐0001‐supinfo.zip. [file ECE3-16-e73247-s001.zip › supinfo file/stigma receptivity IV.png]

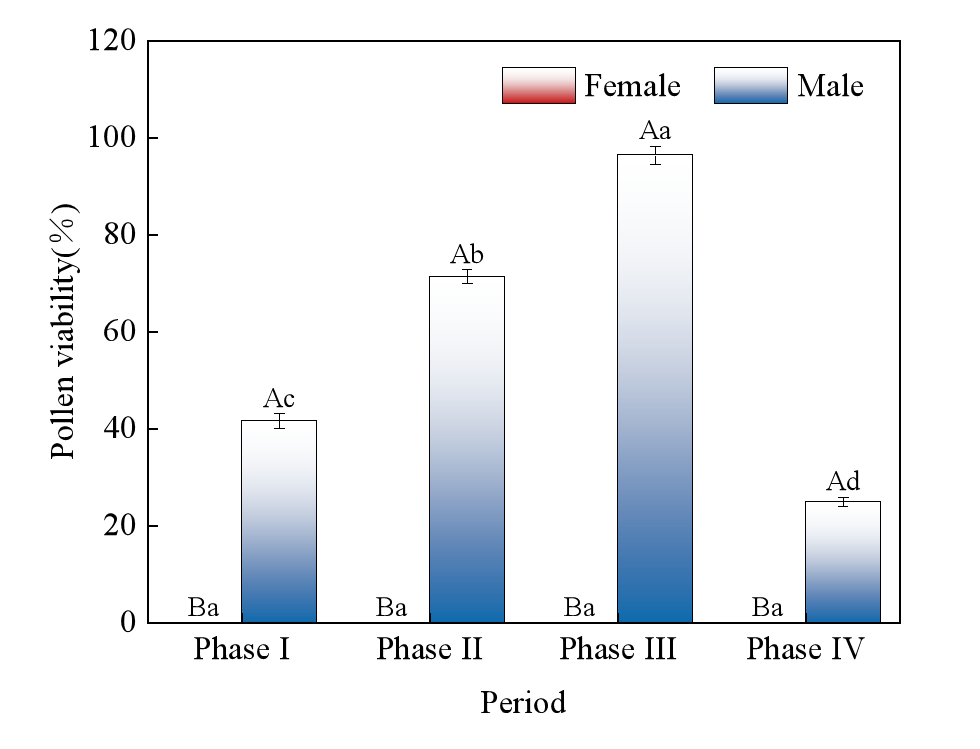

Supplement: Supplementary file 1 — Data S1: ece373247‐sup‐0001‐supinfo.zip. [file ECE3-16-e73247-s001.zip › supinfo file/TTC staining method for detecting pollen viability.png]

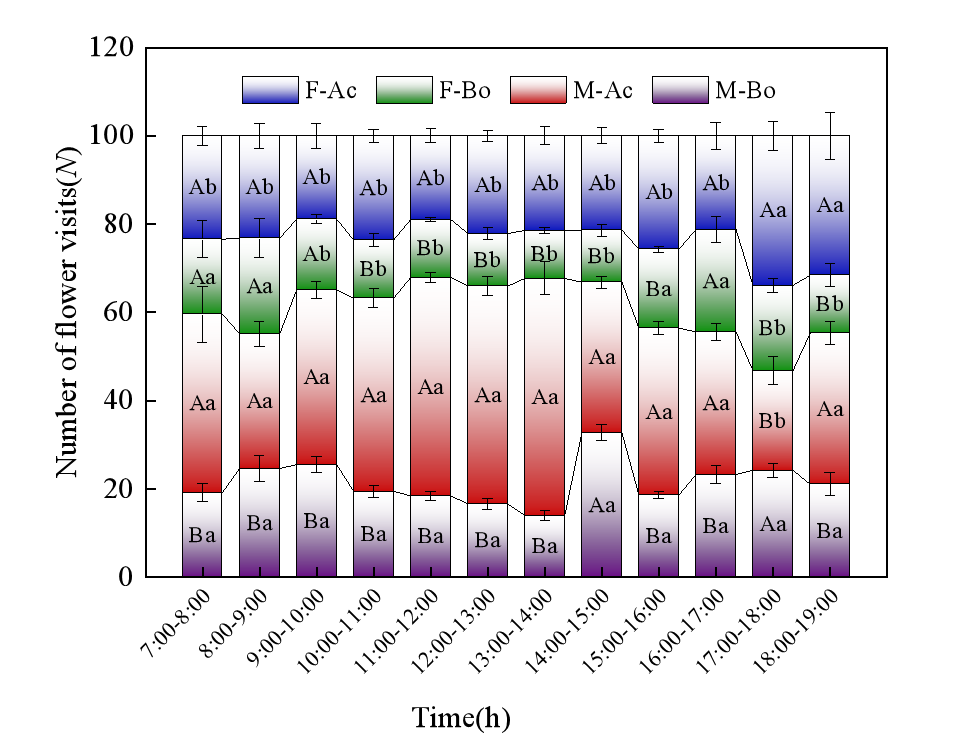

Supplement: Supplementary file 1 — Data S1: ece373247‐sup‐0001‐supinfo.zip. [file ECE3-16-e73247-s001.zip › supinfo file/Visiting frequency of primary pollinator insects.png]

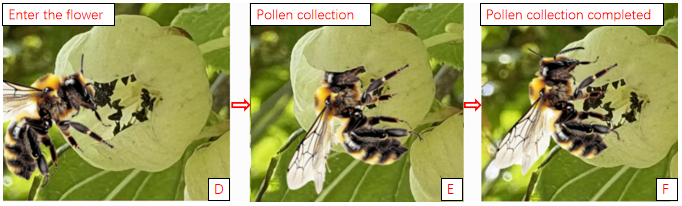

Supplement: Supplementary file 1 — Data S1: ece373247‐sup‐0001‐supinfo.zip. [file ECE3-16-e73247-s001.zip › supinfo file/Visiting process of Apis cerana.png]

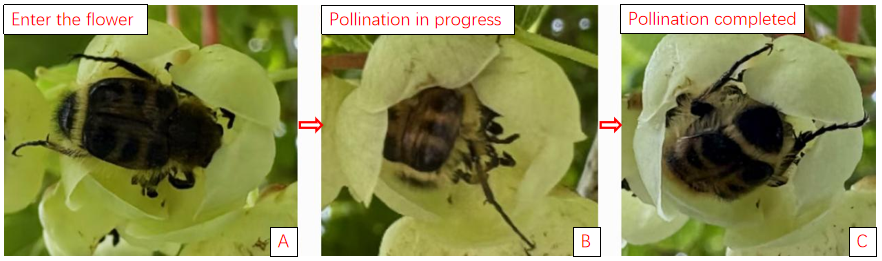

Supplement: Supplementary file 1 — Data S1: ece373247‐sup‐0001‐supinfo.zip. [file ECE3-16-e73247-s001.zip › supinfo file/Visiting process of Bombus.png]
